# Acute aortic occlusion in a patient with chronic paralysis due to spinal cord injury: a case report

**DOI:** 10.1186/s40792-016-0251-5

**Published:** 2016-11-02

**Authors:** Satoshi Yamamoto, Yuriko Yokomizo, Takafumi Akai, Takehiro Chiyoda, Hiroshi Goto, Yukiyoshi Masaki

**Affiliations:** Department of Surgery, Ome Municipal General Hospital, 4-16-5, Higashi Ome, Ome-shi, Tokyo, 198-0042 Japan

**Keywords:** Acute aortic occlusion, Spinal cord injury, Limb ischemia, Peripheral artery disease

## Abstract

Patients with spinal cord injury experience changes in the cardiovascular system and a high morbidity associated with peripheral artery disease. We report a case of acute aortic occlusion in a patient with chronic paralysis due to spinal cord injury. A 65-year-old man with chronic paralysis due to spinal cord injury developed mottling of the right extremity. Because of the complete tetraplegia, the patient had no subjective symptoms. Computed tomography revealed occlusion of the infrarenal abdominal aorta. An emergency thromboembolectomy established adequate blood flow, and the postoperative course was uneventful. The loss of muscle mass might be an advantage in avoiding ischemia reperfusion syndrome. Early detection of acute aortic occlusion and immediate reperfusion are primarily important, but patients with chronic paralysis present a risk of delay in detection, diagnosis, and treatment of acute aortic occlusion because of motor or sensory deficits. Although rare, it is necessary to consider acute aortic occlusion in the case of acute limb ischemia in patients with chronic paralysis due to spinal cord injury.

## Background

Spinal cord injury (SCI) changes the cardiovascular system. SCI causes significant vascular and autonomic dysfunctions, such as blood pressure abnormalities (orthostatic hypotension, autonomic dysreflexia) and rhythm disturbances (bradyarrhythmias, reduced heart rate variability) [1, 2]. Extensive adaptations occur in the peripheral circulation, and the diameter of the arteries in the lower extremities decreases after SCI [3, 4]. Physical inactivity in patients with chronic paralysis due to SCI is relevant to cardiovascular risk factors, including obesity, lipid disorders, metabolic syndrome, and diabetes mellitus [1, 2]. Morbidity related to the cardiovascular diseases is higher in patients with SCI than in ambulatory individuals [1]. Additionally, patients with SCI present a higher risk of peripheral artery disease (PAD) compared with patients without SCI [4, 5]. Meanwhile, the survival rate in patients with SCI has improved [6, 7]. An increased risk of acute arterial occlusion with SCI has not been clarified yet, but SCI might be associated with this condition.

Acute aortic occlusion (AAO) is rare among acute arterial occlusive diseases; however, it is still associated with high mortality and morbidity rates [8–10]. The prevalence of PAD among patients with AAO is reportedly 55 to 70 % [9, 10], and the etiology of AAO has shifted from embolic to thrombotic [10]. To our knowledge, there have been no reports of AAO in patients with chronic paralysis due to SCI. However, patients with chronic paralysis due to SCI, who have high PAD-related morbidity, may present a risk of AAO.

We report a case of AAO in a patient with chronic paralysis due to SCI. The patient consented to the publication of this report.

## Case presentation

**Fig. 1:**
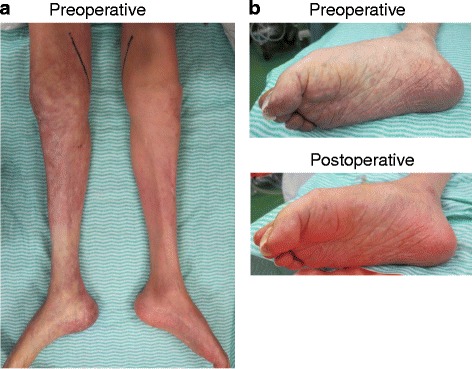
Photographs of the lower extremities. The patient has muscle atrophy in the bilateral lower extremities (**a**). Preoperatively, the skin on the right lower extremity appears pale, mottled, and cyanotic, while ischemic changes of the skin on the left side are mild (**a**). The cyanosis of the right foot disappeared immediately after the thromboembolectomy (**b**)

**Fig. 2:**
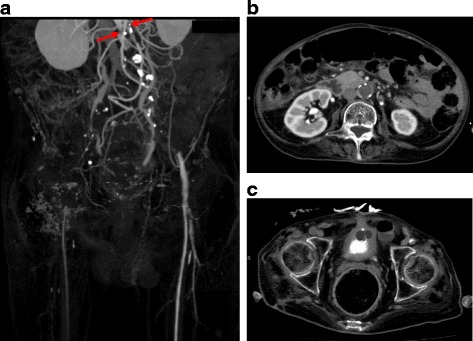
Preoperative computed tomography. The abdominal aorta is occluded below the renal arteries (**a**, *arrows*; **b**). The common, external, and internal iliac arteries are occluded bilaterally (**a**). The infrainguinal vessels were occluded on the right but patent on the left through the collateral vessels (**a**, **c**)

A 65-year-old man, who had complete tetraplegia after SCI to the cervical spine due to a fall at the age of 51 years, developed cyanosis of the right lower extremity and was immediately admitted to our hospital in summer. He had no complaint because of the motor or sensory deficits below the neck. He was a former smoker with impaired glucose tolerance and chronic obstructive pulmonary disease (COPD) but did not have hypertension or hyperlipidemia. On admission, his vital signs were as follows: temperature, 37.5 °C; heart rate, 84 bpm; and blood pressure, 172/104 mmHg. Physical examination revealed mottling of the right lower extremity and loss of the bilateral femoral pulses (Fig. 1). During the examination following admission to the hospital, it was evidenced that the mottling was progressing. Ischemic changes of the skin on the left lower extremity were mild. On the right pedal, the arterial Doppler signal was inaudible while the venous Doppler signal was audible. Blood tests revealed the following values: lactate dehydrogenase (LDH), 213 IU/L; creatine phosphokinase (CPK), 66 IU/L; serum creatinine (Cr) level, 0.13 IU/L; and serum potassium ion concentration, 3.3 mEq/L. The test results for antithrombin III, protein C and S deficiencies, and anticardiolipin antibody were negative. The electrocardiogram showed sinus rhythm. Echocardiography demonstrated normal left ventricular systolic function but did not show left ventricular thrombus or valvular heart disease. An ultrasound scan revealed a hypoechoic thrombus in the distal right external iliac artery. Computed tomography (CT) revealed occlusion of the infrarenal abdominal aorta and bilateral iliac arteries (Fig. 2). The infrainguinal vessels were occluded on the right but patent on the left through the collateral vessels.

Systemic heparin administration was immediately started. After the patient was diagnosed with AAO, he underwent emergent thromboembolectomy using a balloon catheter, which was inserted by means of a bilateral common femoral arteriotomy. An abundant fresh thrombus and a small amount of organized thrombus were obtained from the abdominal aorta and right iliac arteries. The organized thrombus was seen in the proximal portion of the thrombus. The organized thrombus was partial and did not fill the lumen of the arteries. Intraoperative aortography demonstrated that a small amount of thrombus remained in the right common iliac artery and terminal aorta, but the aorta and right iliac arteries did not show stenosis. Meanwhile, a small amount of fresh thrombus was obtained from the left iliac arteries. Although the catheter was passed smoothly through the left iliac arteries, the thrombus of the left iliac arteries was not retrieved completely and the left external iliac artery remained stenotic. The fresh thrombus was also retrieved from the bilateral infrainguinal arteries. Immediately after the revascularization, both foot pulses became palpable.

**Fig. 3:**
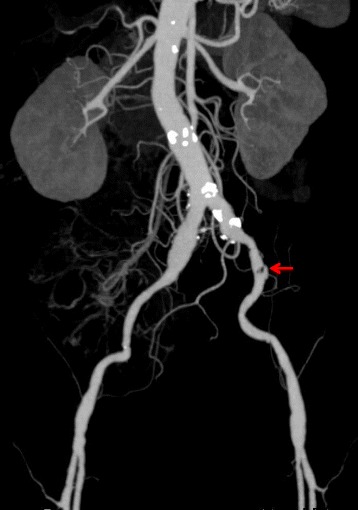
Postoperative computed tomography (3 months after surgery). A small thrombus is visible on the left external iliac artery (*arrow*), but the abdominal aorta and the bilateral common and external iliac arteries are patent. The bilateral internal iliac arteries remain occluded, while the inferior mesenteric artery is patent. The abdominal aorta and the left common iliac artery show calcification, and the right common iliac artery shows dilatation

After revascularization, the patient’s LDH ranged between 159 and 198 IU/L. His CPK levels peaked at 223 IU/L 2 days after revascularization and dropped below 30 IU/L 7 days after revascularization. His serum Cr levels dropped to 0.08 IU/L with hydration 2 days after revascularization and ranged between 0.12 and 0.14 IU/L thereafter. His serum potassium ion concentrations did not exceed 4.1 mEq/L. The patient did not develop myonephrolpathic metabolic syndrome or visceral ischemia. The postoperative course was uneventful, and additional surgical procedures were not required. The patient was prescribed antiplatelet agents after anticoagulation medications. Three months after surgery, postoperative CT revealed that almost all of the remnant thrombus had disappeared, while the abdominal aorta and iliac arteries showed arteriosclerotic changes (Fig. 3). At 2-year follow-up, he remained free of reocclusion.

### Discussion

The occlusion of the aorta and iliac arteries was acute because most of the retrieved thrombus was fresh. Transesophageal echocardiography was not performed because the patient refused the procedure, but he did not have evidence of an embolic source on a limited work-up. Venous thromboembolism is common in patients with SCI [1], but paradoxical embolism was unlikely because he did not have deep vein thrombosis or a patent foramen ovale [11]. CT showed atherosclerotic changes of the aorta and iliac arteries. Therefore, the etiology of AAO in this case was considered thrombotic rather than embolic. Based on the findings of the thrombus and CT scan, we believe that acute-on-chronic aortoiliac artery occlusion developed. Only a small amount of the thrombus in the left iliac arteries was retrieved at the time of surgery because the left iliac arteries were previously occluded. The right common iliac artery showed dilatation with atherosclerotic changes, and the occlusion of the right common iliac artery might induce an acute aorto-right iliac artery occlusion. However, we believe that simultaneous occlusions of the aorto-right iliac arteries and the right femoral artery occurred, rather than occlusion of the aorto-right iliac arteries prior to occlusion of the right femoral artery, because an abundant thrombus was obtained from the aorto-right iliac arteries and the thrombus was as fresh as the thrombus from the infrainguinal arteries. Consequently, we assume that the left iliac artery was previously occluded, and occlusions of the aorto-right iliac artery and right femoral artery followed. Babu et al. reported that the cause of sudden thrombosis in patients with atherosclerotic occlusive disease was precipitated by a low-flow state or by cardiac decompensation [8]. The patient did not have chronic heart failure, and no blood test values showed clear evidence of hypoperfusion. However, we assume that he was dehydrated because the weather was hot and he had a high temperature of 37.5 °C on admission. The patient also was at risk for heatstroke due to infrequent water intake related to chronic paralysis. Dehydration may have contributed to the development of AAO.

In the present case, ischemic pain in the abdomen or limbs was not obvious because of the sensory deficit, and emergence of paresthesia or paralysis from spinal ischemia could not be evaluated because of the preexisting motor or sensory deficits. The left lower extremity showed little ischemic change due to the collaterals induced by the atherosclerotic lesion. Thereafter, only objective symptoms of acute limb ischemia in the right lower extremity, such as pallor, poikilothermia, and pulselessness, were indicative of the diagnosis of AAO. Since subjective symptoms were absent, the absolute time of onset of aortic occlusion was not established. However, the staff in a facility for the handicapped checked his condition on the previous night, and the onset of ischemia was considered to be within 12 h.

The ultrasound scan suggested that the hypoechoic thrombus in the distal right external iliac artery was fresh. On preoperative CT, the thrombus looked homogeneous in density from the aorta to the right external iliac artery. Therefore, we assumed that the occlusion of the aorta and iliac arteries was acute rather than chronic. We supposed that absence of subjective symptoms might mask the severity of AAO. Because patients with AAO have had poor prognosis without surgical treatment [8–10], we opted for surgical treatment. Surgical alternatives for AAO include simple thromboembolectomy, axillofemoral bypass, aortofemoral bypass, and thrombolytic therapy [8]. In the axillofemoral bypass, the risk of graft occlusion was expected to be higher in the patient who lived a sedentary lifestyle compared with the ambulatory individual. The patient with SCI had decrease in the respiratory capacity due to COPD and was not in a good general status for aortofemoral bypass. The effect of thrombolytic therapy was unclear in patients with a massive thrombus. Meanwhile, thromboembolectomy is often associated with difficulty in restoring complete perfusion in patients with in situ thrombosis [9] and poses risks of embolism, dissection, or rupture. If the thromboembolectomy was unsuccessful, we were considering proceeding to an axillofemoral bypass, as other authors stated [9, 10]. However, the simple thromboembolectomy established adequate blood flow.

For the treatment of limb ischemia, the degrees of sensory loss and muscle weakness are useful in diagnosing ischemia that requires emergency surgical treatment [12, 13]. Paralysis is an important indicator of limb viability; limb ischemia with paralysis is considered irreversible, and amputation is necessary. We could not evaluate ischemic motor or sensory dysfunction in the lower extremities because of the preexisting neurological deficit. The onset of ischemia was considered to be within 12 h, but the absolute time was unknown. However, limb ischemia was considered reversible because the venous Doppler signal was audible on the right pedal; the limb ischemia was classified as category IIb on the basis of the clinical categories of acute limb ischemia [12, 13]. Primary amputation was considered acceptable because of the chronic quadriplegia, but simultaneous thromboembolectomy for the infrainguinal arteries and AAO was less invasive than primary amputation. Complete limb revascularization was attempted and achieved by simultaneous thromboembolectomy.

A good outcome was achieved despite the lethal condition in the present case. We considered using continuous hemodiafiltration for ischemia reperfusion syndrome if serum potassium ion concentrations increased drastically, but no postoperative complications developed. The patient’s CPK levels showed significant change after revascularization, but those levels did not exceed normal limits. We believe that CPK and Cr levels were low because the patient had loss of muscle mass due to chronic paralysis, which would make loss of the muscle an advantage in avoiding ischemia reperfusion syndrome.

## Conclusions

Although rare, patients with chronic paralysis due to SCI can develop AAO. Patients with chronic paralysis present a high risk of delay in detection, diagnosis, and treatment of AAO because of motor or sensory deficits. It is necessary to consider AAO in the case of acute limb ischemia in patients with chronic paralysis due to SCI.

## Abbreviations

AAO: Acute aortic occlusion
COPD: Chronic obstructive pulmonary disease
CPK: Creatine phosphokinase
Cr: Serum creatinine
CT: Computed tomography
LDH: Lactate dehydrogenase
PAD: Peripheral artery disease
SCI: Spinal cord injury

## References

[1] Myers J, Lee M, Kiratli J (2007). Cardiovascular disease in spinal cord injury: an overview of prevalence, risk, evaluation, and management. Am J Phys Med Rehabil.

[2] Sezer N, Akkus S, Ugurlu FG (2015). Chronic complications of spinal cord injury. World J Orthop.

[3] de Groot PC, Bleeker MW, van Kuppevelt DH, van der Woude LH, Hopman MT (2006). Rapid and extensive arterial adaptations after spinal cord injury. Arch Phys Med Rehabil.

[4] Bell JW, Chen D, Bahls M, Newcomer SC (2011). Evidence for greater burden of peripheral arterial disease in lower extremity arteries of spinal cord-injured individuals. Am J Physiol Heart Circ Physiol.

[5] Su TW, Chou TY, Jou HJ, Yang PY, Lin CL, Sung FC (2015). Peripheral arterial disease and spinal cord injury: a retrospective nationwide cohort study. Medicine (Baltimore).

[6] van den Berg ME, Castellote JM, de Pedro-Cuesta J, Mahillo-Fernandez I (2010). Survival after spinal cord injury: a systematic review. J Neurotrauma.

[7] Chamberlain JD, Meier S, Mader L, von Groote PM, Brinkhof MW (2015). Mortality and longevity after a spinal cord injury: systematic review and meta-analysis. Neuroepidemiology.

[8] Babu SC, Shah PM, Nitahara J (1995). Acute aortic occlusion-factors that influence outcome. J Vasc Surg.

[9] Surowiec SM, Isiklar H, Sreeram S, Weiss VJ, Lumsden AB (1998). Acute occlusion of the abdominal aorta. Am J Surg.

[10] Crawford JD, Perrone KH, Wong VW, Mitchell EL, Azarbal AF, Liem TK (2014). A modern series of acute aortic occlusion. J Vasc Surg.

[11] Maron BA, Shekar PS, Goldhaber SZ (2010). Paradoxical embolism. Circulation.

[12] Rutherford RB, Baker JD, Ernst C, Johnston KW, Porter JM, Ahn S (1997). Recommended standards for reports dealing with lower extremity ischemia: revised version. J Vasc Surg.

[13] Norgren L, Hiatt WR, Dormandy JA, Nehler MR, Harris KA, Fowkes FG (2007). Inter-society consensus for the management of peripheral arterial disease (TASC II). Eur J Vasc Endovasc Surg.

